# Characterization of RNA Editing in Oxidative and Glycolytic Skeletal Muscles of Yak

**DOI:** 10.3390/biology15010097

**Published:** 2026-01-02

**Authors:** Yilin Shi, Xuemei Wu, Chunnian Liang, Xian Guo, Xiaoming Ma, Ping Yan, Min Chu, Xiaoyun Wu

**Affiliations:** Key Laboratory of Animal Genetics and Breeding on Tibetan Plateau, Ministry of Agriculture and Rural Affairs, Key Laboratory of Yak Breeding Engineering, Lanzhou Institute of Husbandry and Pharmaceutical Sciences, Chinese Academy of Agricultural Sciences, Lanzhou 730050, China; shi_yilin37@163.com (Y.S.); wu_xuemei05@163.com (X.W.); chunnian2006@163.com (C.L.); guoxian@caas.cn (X.G.); maxiaoming@caas.cn (X.M.); pingyanlz@163.com (P.Y.)

**Keywords:** yak, RNA editing, muscle fiber type, transcriptome

## Abstract

Fast-twitch and slow-twitch muscle fibers not only differ in metabolic characteristics and physiological functions but also significantly influence the texture of livestock meat. RNA editing represents an important post-transcriptional regulatory process that can influence both gene expression and the resulting protein function. However, studies on RNA editing events in yak muscle remain limited. This study systematically identified RNA editing events in yak *biceps femoris* (BF, *n* = 3) and *obliquus externus abdominis* (OEA, *n* = 3) using transcriptomic data, discovering 17,713 unique editing sites, most located in non-coding regions. Within coding regions, 3350 sites were detected, with 1195 resulting in non-synonymous amino acid substitutions. Further analysis revealed that 785 sites potentially affected miRNA binding sites, suggesting RNA editing may participate in miRNA-mediated gene regulation. Tukey’s post hoc test (*p* < 0.05) identified 242 sites (involving 170 genes) with significantly different editing levels between BF and OEA. KEGG pathway analysis indicated that genes with differential RNA editing were predominantly associated with pathways involved in muscle fiber type transitions, including the MAPK and calcium signaling pathways. Collectively, this study maps the RNA editing landscape in yak muscle tissue and identifies distinct, fiber-type-specific RNA editing patterns between oxidative and glycolytic muscle fibers, including differences in editing levels and site distributions, supporting a potential association between RNA editing and muscle fiber type transformation.

## Simple Summary

To investigate the role of RNA editing in yak muscle fiber type transformation, this study systematically analyzed RNA editing patterns in yak oxidative muscle fibers and glycolytic muscle fibers tissues by integrating transcriptomic and genomic sequencing data. A total of 17,713 high-confidence RNA editing sites were identified, with 2535 showing significant differences between the two muscle types. Functional enrichment analysis revealed that these differentially edited genes were mainly involved in pathways associated with muscle fiber type regulation, such as the MAPK signaling pathway and calcium signaling pathway. This study provides new molecular evidence for understanding the potential role of RNA editing in the physiological regulation of yak muscle.

## 1. Introduction

Yak (*Bos grunniens*) is endemic to the Qinghai–Tibetan Plateau and adjacent alpine regions, which can adapt to the low oxygen and extremely cold environment. It can use the pasture resources and provide meat, milk, hair, transportation, and fuel for local communities. Their meat is highly nutritious, containing abundant protein and minerals while being relatively low in fat [1]. However, the tenderness of yak meat is generally poor and could not meet the demands of food processing and industrial application [2]. Therefore, improving the meat quality traits is a major priority in the yak industry.

Meat tenderness is largely determined by post-mortem biochemical processes and structural changes in skeletal muscle, which are in turn strongly influenced by the pre-mortem muscle fiber type composition and their associated metabolic properties [3]. Comprising about 40% of human body mass, functions as a key organ in energy production and metabolism. Type I muscle fibers, for instance, are characterized by a high density of mitochondria, abundant myoglobin and elevated triglyceride stores, conferring substantial oxidative capacity and are generally associated with finer texture. In contrast, Type IIB fibers display greater glycogen content and increased ATPase activity [4]. Differences in metabolic activity across skeletal muscle fiber types contribute to a range of human disease states, including Duchenne muscular dystrophy, type 2 diabetes and other disorders of energy metabolism [5]. Meanwhile, in animal husbandry, muscle fiber-type composition exerts a dominant influence on an array of meat quality markers, including muscle pH, color parameters, drip losses, textural tenderness and the level of intramuscular fat [6,7]. Previous research has indicated the proportion of type I and type IIA muscle fiber is negatively correlated with the intramuscular fat content and shearing force of beef [8]. Unraveling the genetic basis underlying the metabolic pattern of muscles could provide the basis for improving meat quality in livestock. Recent studies have identified key genes and signaling pathways that regulate muscle fiber type transformation, including the peroxisome proliferator activated receptor gamma coactivator 1 alpha (*PPARgC1A*), calcineurin, myocyte enhancer factor 2C (*MEF2C*), and the AMP-activated protein kinase signaling pathway [9]. These pathways form intricate networks that affect the expression of fiber-specific genes and metabolic profiles. Therefore, elucidating the muscle fiber type profile in yaks is essential for understanding the biological basis of meat quality and for identifying potential strategies to improve yak meat quality.

RNA editing is a crucial post-transcriptional regulatory mechanism that modifies the genetic information carried by RNA by inserting, deleting, or replacing bases in the primary transcript [10]. The phenomenon of RNA editing was first discovered in the mitochondria of trypanosomes in 1986 and has since been found to occur in the nucleus, chloroplasts, and plasmids as well [11]. In mammals, there are primarily two types of base substitution RNA editing events: A-to-I and C-to-U, with the former being quantitatively dominant. A-to-I editing refers to the process in which adenosine (A) is converted to inosine (I) through deamination at the C6 position, catalyzed by adenosine deaminases acting on RNA (ADARs) [12]. During reverse transcription and translation, inosine is recognized as guanine (G), thus this process is also known as A-to-G editing. On the other hand, C-to-U editing occurs when cytosine (C) is converted to uridine through deamination catalyzed by the Apolipoprotein B mRNA-editing enzyme, catalytic polypeptide-like family of cytidine deaminases [13]. This type of editing mainly occurs in the 3′ untranslated region (UTR). ApoB mRNA editing is the first example of C-to-U editing discovered in mammals, and its editing mechanism has been relatively well characterized. Previous studies have reported that RNA editing plays multiple and diverse functions, including altering amino acid sequences, affecting variable splicing, causing intron retention, and influencing RNA stability [14]. Recent studies have indicated that RNA editing is involved in various biological processes, such as neuronal function, immunity, and disease pathology [15,16,17]. In livestock, RNA editing is also implicated in the development of complex economic traits such as mammary gland development and reproductive performance [18,19]. However, the specific roles of RNA editing in regulating muscle fiber type composition remain largely unexplored.

In this study, we hypothesized that RNA editing represents an additional regulatory layer contributing to molecular differences between oxidative and glycolytic muscle fibers in yak. By systematically characterizing and comparing RNA editing landscapes between these two muscle fiber types, we aimed to determine whether RNA editing exhibits fiber-type–specific patterns and to explore its potential biological relevance in muscle fiber type transformation. Using strand-specific RNA sequencing data from oxidative skeletal muscle (*biceps femoris*, BF) and glycolytic skeletal muscle (*obliquus externus abdominis*, OEA), this study systematically characterized the RNA editing landscape of yak skeletal muscle. In total, 17,713 high-confidence RNA editing sites were identified, including 2535 that exhibited significant differences between the two muscle types. Functional enrichment analysis indicated that these differentially edited genes are primarily involved in pathways associated with muscle fiber type regulation, such as the MAPK and calcium signaling pathways. These findings suggest that RNA editing may serve as an important regulatory mechanism underlying muscle fiber transformation in yaks. This work offers new insights into the formation of muscle fiber types in yaks and the improvement of meat quality.

## 2. Materials and Methods

### 2.1. Data Resource

To investigate RNA editing sites in yak muscle tissue, we downloaded six specific RNA-seq datasets (PRJNA727968) from the NCBI SRA database (https://www.ncbi.nlm.nih.gov/sra?db=sra, accessed on 15 June 2024), obtained from our group’s previous research [20]. This dataset originated from three adult female yaks (5 years old) of similar body weight, with samples collected from their BF and OEA tissues. The cDNA libraries were sequenced using the Illumina HiSeq 2500 platform with a paired-end sequencing strategy (2 × 125 bp).

### 2.2. Whole Genome Sequencing

Blood samples were collected from yak with the same genetic background as the samples used in the RNA-seq study [20]. Genomic DNA was extracted using a commercial DNA extraction kit from Tiangen Biotechnology (Beijing, China), according to the manufacturer’s protocol. DNA library construction was performed using the MGI Easy Universal DNA Library Prep Kit (MGI, Shenzhen, China) and the BGI standard library preparation workflow. Sequencing was conducted by Wuhan Frasergen Bioinformatics Co., Ltd. on the MGISEQ2000 platform (MGI, Shenzhen, China) using paired-end sequencing (2 × 150 bp), with an average sequencing depth of 10×.

### 2.3. Reads Mapping

To generate high-quality reads, sequences containing poly-N stretches, adapter contamination, or low-quality bases were removed. The cleaned WGS reads were aligned to the domestic yak reference genome (LU_Bosgru_v3.0) using BWA mem v0.7.17 [21]. The resulting alignments were sorted with Samtools v1.9 [22], and duplicate reads were subsequently eliminated using the MarkDuplicates function in Picard tools v2.13.2 [23]. Subsequently, clean reads from the RNA-seq data were independently aligned to the same reference genome (LU_Bosgru_v3.0) using HISAT2 v2.1.0 [24]. The resulting SAM files were transformed into BAM format and subsequently sorted using SAMtools v1.9 [22].

### 2.4. RNA Editing Detection

To investigate the genomic characteristics of RNA editing in yak muscles with different fiber types, we identified RNA editing events using the REDItoolDnaRna.py module in REDItools v1.0.4 [25], which enables direct comparison between RNA-seq and DNA-seq data to detect RNA–DNA differences (RDDs). To minimize false-positive signals and reduce the influence of genomic polymorphisms, RNA variants overlapping with DNA variants detected in the WGS data were excluded from downstream analyses. Filtering parameters were set based on previous studies with minor adjustments [26]. In addition, only RNA editing sites detected in at least two biological replicates were retained as high-confidence editing events. Finally, retained RNA editing sites were annotated using SnpEff v4.3t [27] based on Ensembl gene annotations (release 90).

### 2.5. Validation of RNA Editing Sites Through Sanger Sequencing

To verify the credibility of the RNA editing sites detected in this study, two candidate sites were randomly selected for experimental validation using polymerase chain reaction (PCR). Six samples previously used for RNA sequencing in this study were selected for validation. Total RNA was isolated using the Animal Tissue RNA Isolation Kit (ZDGSY, Beijing, China) in strict accordance with the manufacturer’s protocol, while genomic DNA was extracted with the corresponding Animal Tissue Genomic DNA Kit (ZDGSY, Beijing, China). Complementary DNA was synthesized from RNA templates using the PrimeScript™ RT Reagent Kit with gDNA Eraser (TaKaRa, Dalian, China). Equal quantities of the resulting cDNA products were combined and used as templates for PCR amplification. Primer sequences are provided in Table S1. Each PCR reaction was carried out in a total volume of 25 μL, consisting of 4 μL pooled cDNA, 1 μL of each primer, 6.5 μL GoTaq^®^ Green Master Mix (Promega, Madison, WI, USA), and 12.5 μL nuclease-free water. The PCR program began with an initial denaturation at 95 °C for 2 min, followed by 35 cycles consisting of 30 s at 95 °C for denaturation, 30 s at 68 °C for primer annealing, and 30 s at 72 °C for extension, concluding with a final elongation step at 72 °C for 5 min. The amplified products were subsequently subjected to Sanger sequencing for validation.

### 2.6. Analysis of the Impact of RNA Editing on miRNA Regulation

RNA editing events occurring within mRNA 3′UTRs were assessed for their potential influence on miRNA binding. Both RNAhybrid v2.1.2 (parameters: −b 1 −c −f 2,8 −m 100,000 −u 1 −v 1 −e −10) [28] and miRanda v3.3a (sc 140, en −10, scale 4, strict) [29] were applied to predict miRNA–mRNA interactions using edited sequences as well as their corresponding unedited counterparts. Only miRNA–target interactions concurrently identified by both algorithms were retained for subsequent analyses. Interactions detected exclusively in edited transcripts were defined as gains of miRNA binding, whereas those present only in the reference sequences were classified as losses of miRNA binding following editing.

### 2.7. Pairwise Comparison of AG Sites Between Groups

The RNA editing level for a given sample at an RNA editing site was calculated as the ratio of reads supporting the edited base to the total number of reads detected at that site. To identify RNA editing sites potentially involved in muscle fiber type conversion, differences in editing levels between BF and OEA groups were evaluated using Tukey’s Honest Significant Difference test. Sites with a *p*-value < 0.05 were considered significantly.

### 2.8. Enrichment Analysis

Genes harboring RNA editing sites within exonic regions were selected for downstream functional annotation. To investigate the biological roles of these candidate genes, Gene Ontology (GO) enrichment analysis was conducted using the G:Profiler online platform [30]. In addition, pathway enrichment analysis based on the Kyoto Encyclopedia of Genes and Genomes (KEGG) database was performed with KOBAS 3.0 software [31] via a hypergeometric statistical framework. GO terms and KEGG pathways with *p* values below 0.05 were regarded as significantly enriched.

### 2.9. Conservation Analysis of RNA Editing Sites

To identify evolutionarily conserved A-to-I RNA editing events, editing sites detected in this study were compared with known human RNA editing sites retrieved from the REDIportal database [32]. A total of 4,627,557 validated human editing sites were included in the conservation analysis. For each yak editing site, 50 bp upstream and downstream flanking sequences were extracted from the domestic yak reference genome (LU_Bosgru_v3.0) and aligned against corresponding flanking regions of human A-to-I editing sites using NCBI BLAST (https://blast.ncbi.nlm.nih.gov/Blast.cgi, accessed on 15 July 2024). Editing sites were considered highly conserved when alignments met the criteria of an Expect (E) value ≤ 1 × 10^−5^ and a sequence identity of at least 85%. The E value reflects the probability of obtaining a comparable alignment by random chance during database searching.

## 3. Results

### 3.1. Identification of RNA Editing Site in BF and OEA Muscles

**Figure 1:**
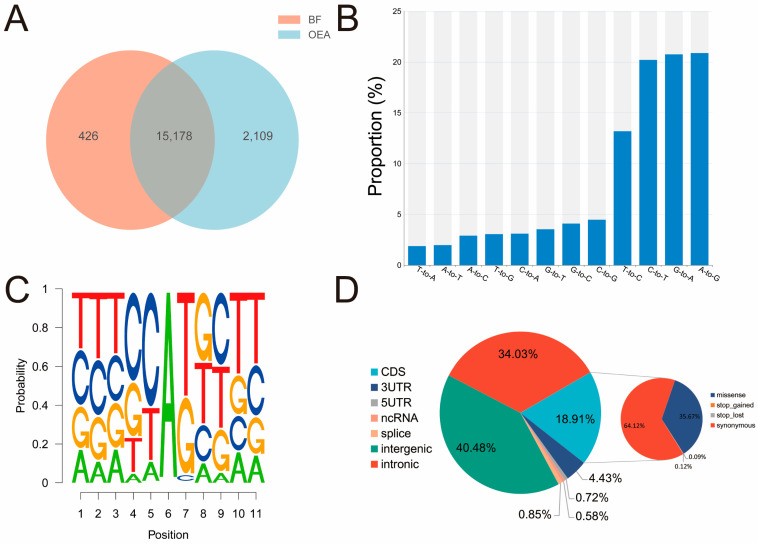
Overview of RNA editing in yak BF and OEA muscles. (**A**) Shared and unique editing sites; (**B**) Distribution of different categories of RNA editing events; (**C**) Neighbor sequence preferences of A-to-G editing; (**D**) Annotation of RNA editing sites.

**Figure 2:**
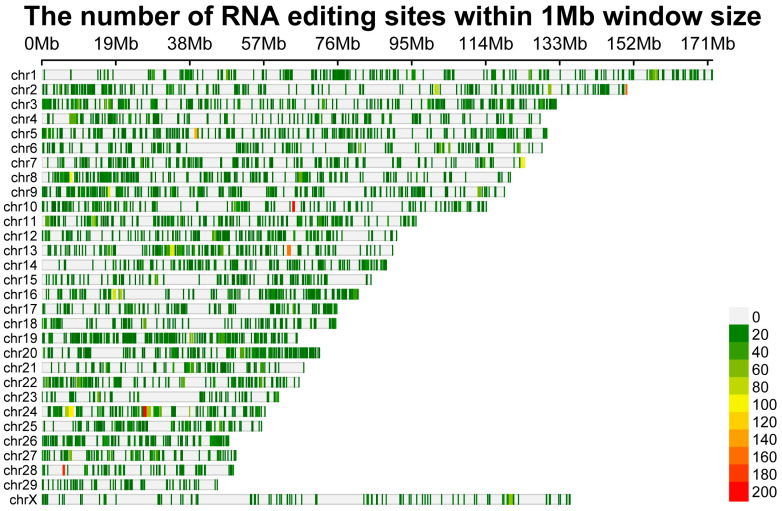
Genomic distribution of RNA editing sites in yak BF and OEA muscles.

After removing adapter sequences and filtering out low-quality reads from the raw sequencing data, we obtained approximately 273.3 million and 264.3 million high-quality clean reads from the BF and OEA muscle samples, respectively. Following rigorous standardized filtering to exclude false positives, a total of 17,713 unique RNA editing sites were identified (Table S2). Although OEA muscles harbored a higher number of unique RNA editing sites than BF muscles (2109 vs. 426), this difference did not reach statistical significance (paired t-test, *p* = 0.153) (Figure 1A). A total of 15,178 editing sites were shared between the two muscle types. Further analysis revealed differential distribution of RNA editing sites across chromosomes (Figure 2), with the highest number occurring on chromosome 3 (1034 sites), followed by chromosome 24 (1015 sites). Chromosome 29 exhibited the lowest number of editing sites at only 171. Two RNA editing sites were randomly chosen for experimental validation using PCR followed by Sanger sequencing. Comparison between genomic DNA and cDNA sequences revealed nucleotide differences at the predicted editing positions. Both partially edited (heterozygous) and fully edited cDNA signals were observed, supporting the predicted RNA editing events (Figure S1).

### 3.2. Sequence Preference and Annotation of RNA Editing Sites in Muscle Tissue

Among the 12 detected RNA editing events, approximately 41.09% corresponded to A-to-G (20.88%) and C-to-T (20.21%) substitutions, representing typical A-to-I and C-to-U RNA editing types (Figure 1B). To further investigate the sequence context features of A-to-G editing events, we analyzed the composition of the 5 bases upstream and downstream of the editing site. Results revealed a significantly reduced frequency of G at the first base upstream of the editing site, while the proportion of G at the first base downstream was significantly elevated (Figure 1C). This sequence preference aligns with the classic substrate recognition features of ADAR enzymes in mammals. Annotation of RNA editing sites revealed that all editing events overlapped with 5422 annotated genes, distributed across seven gene regions: intergenic regions, introns, Coding DNA Sequence (CDS), 3′UTRs, 5′UTRs, non-coding RNAs, and splice sites (Figure 1D; Table S3). The majority of RNA editing events were found in intergenic regions (40.48%), followed by intronic regions (34.03%). Previous studies have shown that RNA editing events occurring within coding sequences can lead to nonsynonymous amino acid substitutions and may alter the structure, stability, or interactions [12]. Therefore, we speculate that the 1195 coding-region RNA editing events identified in this study may potentially affect protein structure.

### 3.3. Comparative Analysis of RNA Editing Between Yak and Human

To investigate the conservation of RNA editing sites between humans and yak, we performed homology comparisons of sequences upstream and downstream of each RNA editing site using nucleotide BLAST. By setting stringent screening thresholds (homology > 85%, E-value < 1 × 10^−5^), we identified a total of 9 conserved A-to-I type RNA editing sites (Table S4). Among these conserved sites, one within the *SON* gene is located within the CDS. Editing at this site could potentially result in amino acid substitution (missense mutation), suggesting potential functional conservation of this site across different species.

### 3.4. Impact of RNA Editing on MiRNA Regulation

**Figure 3:**
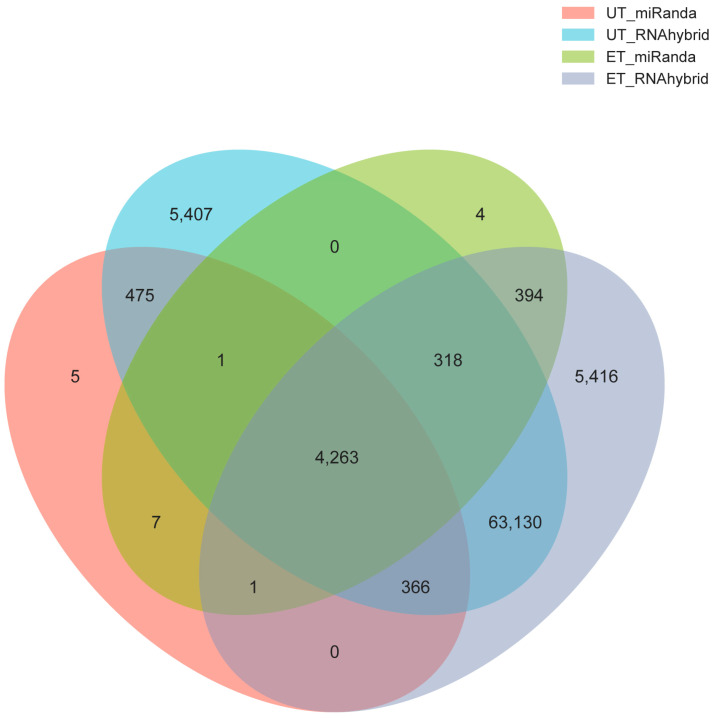
RNA editing sites affecting miRNA binding in yak muscles. In the figure, ET represents newly generated sites, and UT represents abolished sites.

RNA editing may influence miRNA-mRNA interactions by altering miRNA binding sites. This study identified 785 RNA editing events within the 3′UTR region that could potentially affect miRNA binding capacity. We predicted miRNA binding targets at these sites using RNAhybrid and miRanda on the reference sequences. Results revealed that these RNA editing events newly generated 394 miRNA binding sites while disrupting 475 existing sites (Figure 3; Table S5). Overall, 295 genes may be affected by RNA editing-mediated changes in miRNA binding, suggesting that RNA editing may play a crucial role in post-transcriptional regulation between different muscle fiber types through modulating miRNA–mRNA interactions. Further GO and KEGG enrichment analysis of these target genes revealed significant enrichment in 806 GO terms, including 567 biological process (BP), 117 cellular component (CC), and 122 molecular function (MF) entries (Figure S2). These primarily involved functional categories such as biological process, biological regulation, and cellular process. Additionally, 221 KEGG pathways were significantly enriched, including signaling pathways closely related to muscle fiber type transformation, such as the Notch signaling pathway.

### 3.5. Analysis of Differential RNA Editing in BF and OEA Muscles

**Figure 4:**
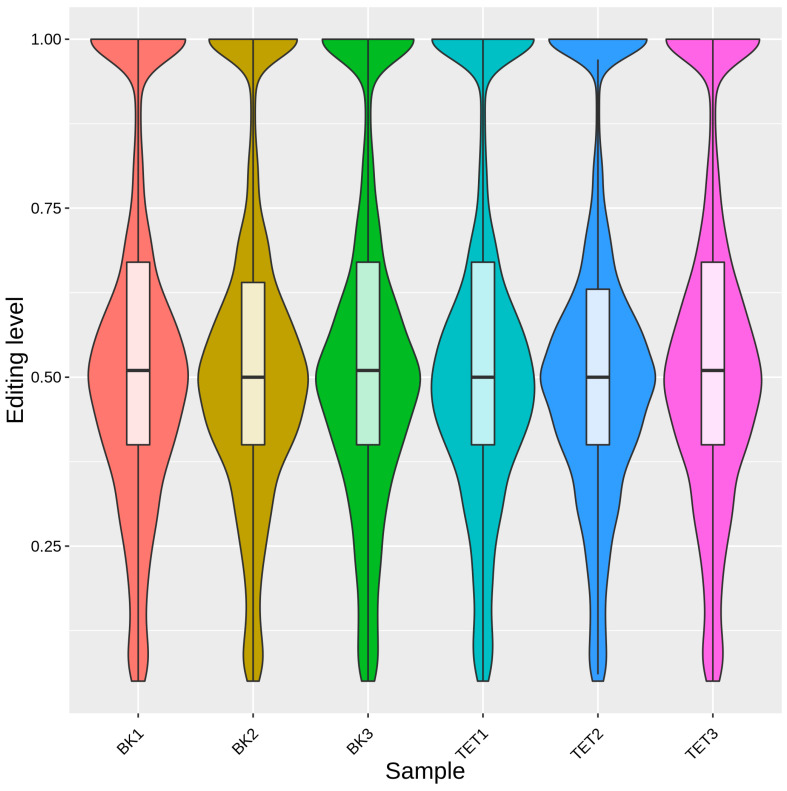
Distribution of RNA editing levels across yak BF and OEA muscle samples.

**Figure 5:**
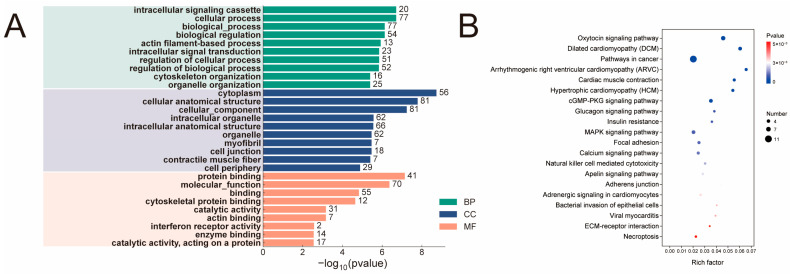
Functional enrichment analysis of genes exhibiting differential RNA editing between BF and OEA muscle tissues. (**A**) GO categories. (**B**) KEGG pathways. The Rich Factor represents the ratio of genes with differential RNA editing to the total number of genes annotated within each functional term or pathway.

The average RNA editing levels across all samples ranged between 0.54 and 0.56, indicating overall high RNA editing level in yak muscle tissue (Figure 4). Differential editing analysis identified a subset of RNA editing events with tissue-specific patterns, suggesting localized yet potentially functionally significant RNA editing regulation between different muscle fiber types. To further identify RNA editing events associated with muscle fiber type transformation, this study compared editing level of each event between BF and OEA samples using Tukey’s post hoc significance test (*p* < 0.05). Results identified 242 differentially edited events distributed across 170 genes (Table S6). GO enrichment analysis showed these genes were significantly enriched in 508 functional entries (q-value < 0.05), including 397 BP, 62 CC, and 49 MF. Enrichment was primarily observed in functional categories such as intracellular signaling cassettes, cellular processes, and biological processes (Figure 5A; Table S7). KEGG pathway analysis further indicated that these differentially edited genes were significantly enriched in multiple signaling pathways closely related to muscle fiber type regulation, such as the MAPK signaling pathway and calcium signaling pathway (Figure 5B; Table S8). This suggests that RNA editing may participate in the muscle fiber type transformation of yak muscle by regulating key signaling pathways.

## 4. Discussion

RNA editing is a crucial post-transcriptional regulatory mechanism in eukaryotes that modifies gene expression and function by altering RNA sequences. However, current research on RNA editing events in yak remains limited. To explore the potential regulatory role of RNA editing in yak skeletal muscle, this study systematically identified RNA editing sites in two yak muscle tissues (oxidative muscle fibers and glycolytic muscle fibers) at the genome-wide level. We further analyzed their distribution patterns, functional enrichment, and potential associations with miRNA binding, aiming to provide new molecular insights into the post-transcriptional regulatory mechanisms underlying muscle fiber composition in yak.

This study identified a total of 17,713 RNA editing events in yak BF and OEA muscle tissues. Results revealed that the number of editing events in OEA muscle was significantly higher than in BF muscle, with OEA muscle harboring 1683 more unique editing sites than BF muscle, suggesting higher RNA editing activity in OEA muscle. Our study revealed differences in RNA editing regulation between muscle fiber types, which may result from variations in ADAR activity, transcript abundance, or other cellular factors [33]. We observed distinct RNA editing patterns between oxidative and glycolytic muscle tissues, likely reflecting the influence of these factors. A detailed categorization of RNA editing events showed that the two canonical forms, A-to-I and C-to-U substitutions, together comprised 41.09% of all detected edits, supporting the overall reliability of the RNA editing dataset generated in this study. To further investigate the conservation of yak RNA editing events, their upstream and downstream sequences were aligned against the human RNA editing database. The results revealed a low degree of overlap in RNA editing sites between yak and human, reflecting the largely species-specific nature of RNA editing reported in previous studies [34,35]. Our results confirm that RNA editing events exhibit a high degree of species specificity. Such differences likely arise from the combined effects of genomic sequence variation, differential expression levels of editing enzymes (e.g., the ADAR family), and substrate recognition sequence preferences across species [36]. Notably, we identified a conserved nonsynonymous RNA editing event in the SON gene. SON encodes a highly conserved RNA splicing factor that plays a critical role in pre-mRNA processing and is essential for proper cell differentiation and development [37]. The presence of a conserved nonsynonymous editing event in such a key splicing regulator may suggest that this RNA editing event has the potential to exert broad effects on muscle-related regulatory networks.

Compared to most previous RNA editing studies, the RNA editing sites identified in this study were more frequently distributed in intergenic regions (40.48%) and intron regions (34.03%), while the proportion of editing sites in CDS regions also significantly increased (18.91%). Previous studies generally suggest that editing sites within CDS regions typically account for less than 10% [34,38], indicating that specific RNA editing regulatory features may exist in yak muscle tissue. Editing events occurring within CDS regions can lead to non-synonymous mutations, directly affecting protein structure and function [39]; whereas editing events in introns or intergenic regions may indirectly regulate gene expression by modulating alternative splicing or affecting post-transcriptional processing [40,41]. Thus, this distribution pattern of editing sites indicates that RNA editing in yak muscle tissue not only participates in post-transcriptional regulation but may also exert direct effects on protein function.

Since RNA editing events within CDS regions can directly alter protein sequences, their biological significance is generally more pronounced than that of editing in non-coding regions. Recoding editing is considered one of the most functionally potent types of RNA editing, capable of modulating protein function through non-synonymous substitutions and thereby promoting proteomic diversity [42]. Therefore, this study further conducted an in-depth analysis of RNA editing sites causing non-synonymous substitutions within CDS regions to investigate their distribution and potential functional impact on genes associated with muscle fiber composition in yak. A total of 1195 recoding RNA editing sites were identified across 877 genes, with some sites located in key genes closely associated with muscle fiber formation. *MYH3* encodes an embryonic type myosin heavy chain (MYHC) that is highly expressed during embryonic to neonatal stages. In adult skeletal muscle, it is transiently re-expressed only during regeneration following injury or pathological conditions [43]. Studies indicate that *MYH3* knockout leads to a significant reduction in slow-twitch fiber proportion in adult mouse muscle [44]. *MYH4*, also a member of the MYHC family, is a typical fast-twitch fiber-associated gene, and its deletion reduces the number of IIB fibers [45]. These findings suggest that the recoding RNA editing events identified in this study may participate in the formation and transition of yak muscle fiber types by regulating the expression or function of such key genes. Additionally, the study found that RNA editing in the 3′UTR may regulate target gene expression by altering miRNA binding sites or affecting the miRNA sequence itself [16]. A total of 295 genes potentially affected by 3′UTR editing were identified. Previous studies have shown that HIF-2α plays a role in the regulation of skeletal muscle fiber type composition, and its expression is closely associated with enhanced glycolytic muscle fiber characteristics [46]. RNA editing events identified in the 3′ UTR of EPAS1 were predicted to influence miRNA–mRNA interactions, suggesting a potential post-transcriptional mechanism by which RNA editing may modulate HIF-2α–associated regulatory pathways and muscle fiber-type–specific metabolic and functional features. KEGG enrichment analysis revealed significant enrichment of these genes in pathways including Notch signaling, starch and sucrose metabolism, and glycerophospholipid metabolism. These pathways are closely linked to the composition and functional properties of skeletal muscle fibers—such as the proportion of slow-twitch (oxidative) versus fast-twitch (glycolytic) fibers, their metabolic capacity, and structural characteristics. [47,48,49].

This study identified 242 differentially edited sites across the BF and OEA groups, involving 170 genes. KEGG enrichment analysis revealed significant enrichment of two key pathways associated with muscle fiber type conversion: the MAPK signaling pathway and the Calcium signaling pathway. The MEK1-ERK2 signaling pathway, a critical component within MAPK signaling, is recognized as fundamental for the conversion of fast-twitch to slow-twitch fibers in both adult skeletal muscle and during development [50]. Concurrently, the Calcium signaling pathway plays a crucial role in the formation and shaping of muscle fiber types. *PGC-1α* not only serves as an upstream regulator of calcium signaling but also drives muscle fiber type conversion [51]. The genes corresponding to the differentially edited sites include key regulators such as *PPARGC1A*, *MEF2C*, and *MYH4*, which play crucial roles in muscle fiber type regulation and muscle function. *PPARGC1A*, a transcription coactivator, is known to promote slow-twitch fiber formation and serves as a critical factor in regulating muscle fiber type determination [52]. Research has shown that knocking out *PPARGC1A* in mouse skeletal muscle results in a marked shift in muscle fiber types, transitioning from oxidative Type I and Type IIa fibers to Type IIx and Type IIb fibers [53]. *MEF2C* belongs to the MEF2 transcription factor family and is a key regulator in skeletal and cardiac muscle transformation [54]. *MEF2C* skeletal muscle-specific knockout mice exhibit a marked reduction in slow-twitch fiber proportion, further demonstrating *MEF2C*’s crucial role in muscle fiber type determination [55]. *MYH4* is a myosin heavy chain specifically expressed in fast-twitch fibers, playing a vital role in the formation of fast-contracting muscle fibers [56]. Studies indicate that *MYH4* knockout mice lack IIB-type fibers and their associated proteins, demonstrating the gene’s essential function in fast-twitch fibers [45]. Taken together, these findings indicate a correlation between differential RNA editing patterns and muscle fiber type transformation. While RNA editing may modulate the expression of key genes through post-transcriptional mechanisms in adult skeletal muscle, the present study does not establish a causal relationship between RNA editing and muscle fiber type determination. Future studies incorporating experimental validation will be essential to confirm the predicted effects of RNA editing on miRNA binding and protein function.

## 5. Conclusions

This study systematically analyzed RNA editing events in yak BF and OEA muscle tissues, identifying a total of 17,713 high-confidence editing sites. Differential editing analysis identified multiple genes closely associated with skeletal muscle function and fiber type composition, suggesting that RNA editing may influence the formation of fast and slow muscle fiber types by modulating the expression of key genes. This study not only enriches the yak RNA editing site resource library but also provides new theoretical support for further elucidating the molecular regulatory mechanisms and physiological adaptability of yak muscle.

## Data Availability

The original contributions presented in the study are included in the article/Supplementary Material, further inquiries can be directed to the corresponding authors.

## Supplementary Materials

The following supporting information can be downloaded at: https://www.mdpi.com/article/10.3390/biology15010097/s1, Figure S1: Validation of RNA editing sites by PCR and Sanger sequencing; Figure S2: Functional enrichment analysis of genes potentially affected by RNA editing-mediated changes in miRNA binding in yak muscles. (A) GO enrichment terms; (B) KEGG pathway enrichment terms; Table S1: Primers used in the Sanger sequencing; Table S2: RNA editing sites in yak skeletal muscles composed of different fiber types; Table S3: Annotation of RNA editing sites in BF and OEA yak muscles; Table S4: Conserved RNA editing sites identified between yak and human genomes; Table S5: Predicted miRNA binding site gains and losses caused by RNA editing events in yak; Table S6: Differentially RNA editing events between BF and OEA tissues; Table S7: GO enrichment results of genes containing differentially edited RNA editing sites between BF and OEA muscles; Table S8: KEGG pathway enrichment results of genes containing differentially edited RNA editing sites between BF and OEA muscles.

## Abbreviations

The following abbreviations are used in this manuscript:

BF	Biceps femoris
OEA	Obliquus externus abdominis
MyHC	Myosin heavy-chain
PPARgC1A(PGC-1α)	Proliferator Activated Receptor Gamma Coactivator 1 Alpha
MEF2C	Myocyte Enhancer Factor 2C
ADAR	Adenosine deaminases acting on RNA
A	Adenosine
I	Inosine
G	Guanine
C	Cytosine
T	Thymine
U	Uracil
UTR	Untranslated region
GO	Geneontology
KEGG	Kyoto Encyclopedia of Genes and Genomes
BLAST	Basic Local Alignment Search Tool
E	Expect
CDS	Coding DNA Sequence
BP	Biological process
CC	Cellular component
MF	Molecular function
