# Supplementary figures and images for: Characterization of RNA Editing in Oxidative and Glycolytic Skeletal Muscles of Yak
Source: Biology (Basel). 2026 Jan 2;15(1):97. doi: 10.3390/biology15010097 (PMC12784926; doi:10.3390/biology15010097)

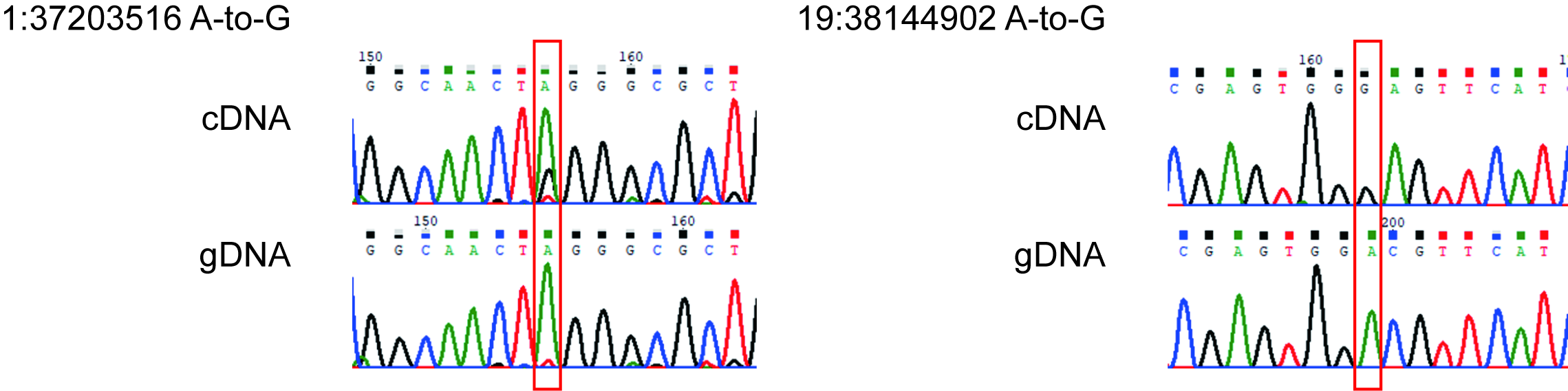

Supplement: Supplementary file 1 [file biology-15-00097-s001.zip › Figure S1.tif]

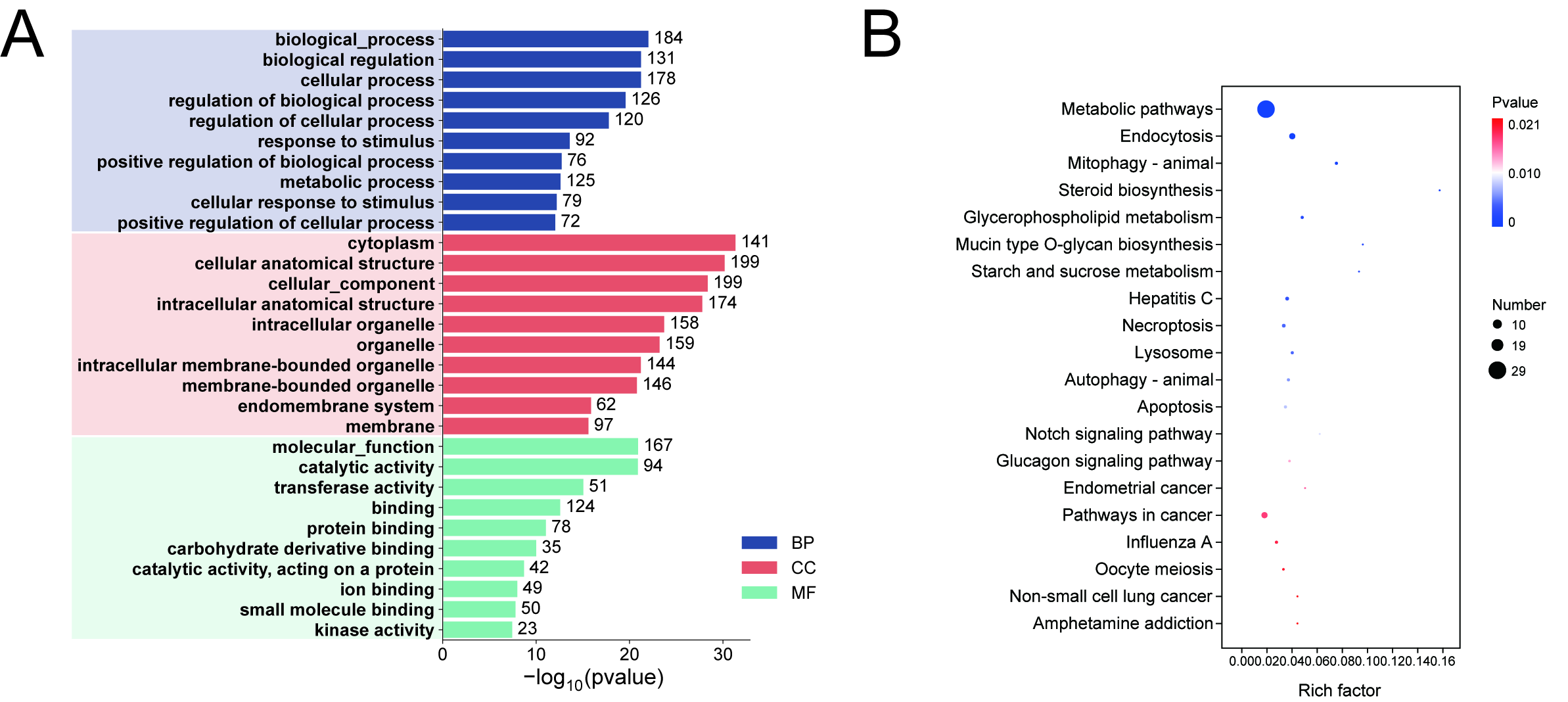

Supplement: Supplementary file 1 [file biology-15-00097-s001.zip › Figure S2.tif]
